# Bio‐Inspired Skin Reconstruction Using Intraoperative Autologous Epidermal Stem Cell–Enhanced Composite Grafts: A Randomized Trial

**DOI:** 10.1002/mco2.70973

**Published:** 2026-09-06

**Authors:** Zhicheng Hu, Hao Yang, Peng Wang, Xiaoling Cao, Peng Wang, Bing Tang, Hailin Xu, Zhiyong Wang, Shaobin Huang, Jian Zhang, Chunli Xue, Qiang Zhou, Xiaohui Li, Honglin Wu, Yi Zhang, Julin Xie, Jiayuan Zhu

**Affiliations:** ^1^ Department of Burn and Wound Repair First Affiliated Hospital of Sun Yat‐Sen University Guangzhou China; ^2^ Department of Burns and Plastic Surgery Linfen Central Hospital Linfen China; ^3^ Department of Dermatology Dermatology Hospital of Southern Medical University Guangzhou China; ^4^ Department of Joint Surgery The Third Affiliated Hospital of Guangzhou Medical University Guangzhou China; ^5^ Department of Plastic and Cosmetic Surgery Sixth Affiliated Hospital of Sun Yat‐Sen University Guangzhou China; ^6^ Department of Plastic and Reconstructive Surgery Sun Yat‐Sen Memorial Hospital of Sun Yat‐Sen University Guangzhou China; ^7^ Department of Burn Surgery Huizhou Central People's Hospital Huizhou China; ^8^ Department of Burn and Plastic Surgery The First People's Hospital of Zhaoqing Zhaoqing China; ^9^ Department of Plastic Surgery & Burn Surgery Shenzhen Hospital Southern Medical University Shenzhen China; ^10^ Department of Burn and Plastic Surgery Department of Wound Repair Surgery Affiliated Hospital of Nantong University Nantong China

**Keywords:** cytokines, epidermal stem cells, regenerative medicine, scar quality, tissue‐engineered skin, wound healing

## Abstract

Full‐thickness wounds remain a major clinical challenge, and tissue‐engineered skin using epidermal stem cells (EpiSCs) offers a promising strategy for bio‐inspired reconstruction. In this randomized controlled trial, 232 patients with full‐thickness defects (≥ 9 cm^2^) received either standard treatment (acellular dermal matrix [ADM] + split‐thickness skin graft [STSG], *n* = 112) or cell therapy (ADM + STSG + EpiSC suspension, *n* = 120). Autologous EpiSCs were prepared intraoperatively in a single step, avoiding prolonged in vitro expansion. The primary outcome was 6‐month scar quality assessed by the Vancouver Scar Scale (VSS), while secondary outcomes included 2‐week healing and 3‐month recurrence. Compared with standard treatment, cell therapy produced significantly lower total VSS scores (median 3.0 vs. 6.0, *p* < 0.001) and improved complete wound healing at 2 weeks (80/120 [66.7%] vs. 61/112 [54.5%]; adjusted odds ratios [OR] = 1.87, 95% confidence interval [CI] 1.06–3.28, *p* = 0.029). Complete healing at 2 weeks was associated with reduced 3‐month recurrence (adjusted OR = 0.26, 95% CI 0.09–0.75, *p* = 0.012). These findings support intraoperative EpiSC‐enhanced composite grafting as an effective and clinically translatable approach for rapid closure and improved long‐term scar outcomes.

## Graphical abstract

Full‐thickness skin defects remain a major reconstructive challenge. This randomized trial demonstrates that single‐stage composite grafting using intraoperatively prepared autologous EpiSC‐enriched suspensions is feasible. EpiSC augmentation improves complete wound healing and yields superior 6‐month scar outcomes compared with standard grafting, providing clinical evidence for a practical, cell‐based strategy for bio‐inspired skin reconstruction.

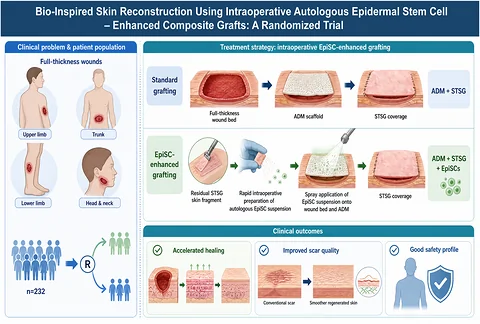

## 1 Introduction

Full‐thickness skin wounds, resulting from trauma, burns, surgery, or chronic diseases such as diabetes, are reported to pose ongoing challenges in clinical practice. These injuries, involving all layers of the skin, often result in both functional impairment and cosmetic concerns [1, 2]. Currently, skin grafting is the most widely used strategy for managing full‐thickness wounds. Traditional grafting methods—including split‐thickness, medium‐thickness, full‐thickness, and composite grafts—each have benefits and limitations [3]. While split‐thickness grafts offer high engraftment and reduced donor site morbidity, they are prone to contraction [4]. Medium‐ and full‐thickness grafts provide better durability and appearance but involve greater donor site trauma [5]. Composite grafts reduce donor damage by integrating artificial dermal matrices, yet they suffer from delayed vascularization and lower initial success rates [6]. Overall, current techniques struggle to balance aesthetic and functional outcomes with donor site preservation [7], highlighting the need for novel therapies that minimize donor site injury while better replicating native skin structure and function.

In recent years, tissue‐engineered skin has emerged as a promising strategy for wound repair, gaining significant attention in regenerative medicine [8, 9, 10]. This approach typically combines scaffold structures with seed cells to address challenges such as delayed vascularization, which is a common issue with conventional composite grafts [11]. The incorporation of viable cells is believed to endow tissue‐engineered skin with functional properties that closely resemble those of native tissue. Among the various cellular options, epidermal stem cells (EpiSCs) have shown particular promise, as they may enhance wound healing outcomes while simultaneously reducing immune rejection and avoiding the ethical concerns associated with embryonic or allogeneic cell sources [12, 13, 14, 15, 16]. However, current tissue‐engineered skin is often viewed as technically complex, expensive, and time consuming, limiting their clinical application [9, 17]. Overcoming these challenges, particularly through rapid and cost‐effective intraoperative isolation of EpiSCs, is essential for broader clinical adoption and the advancement of bio‐inspired skin reconstruction.

**FIGURE 1:**
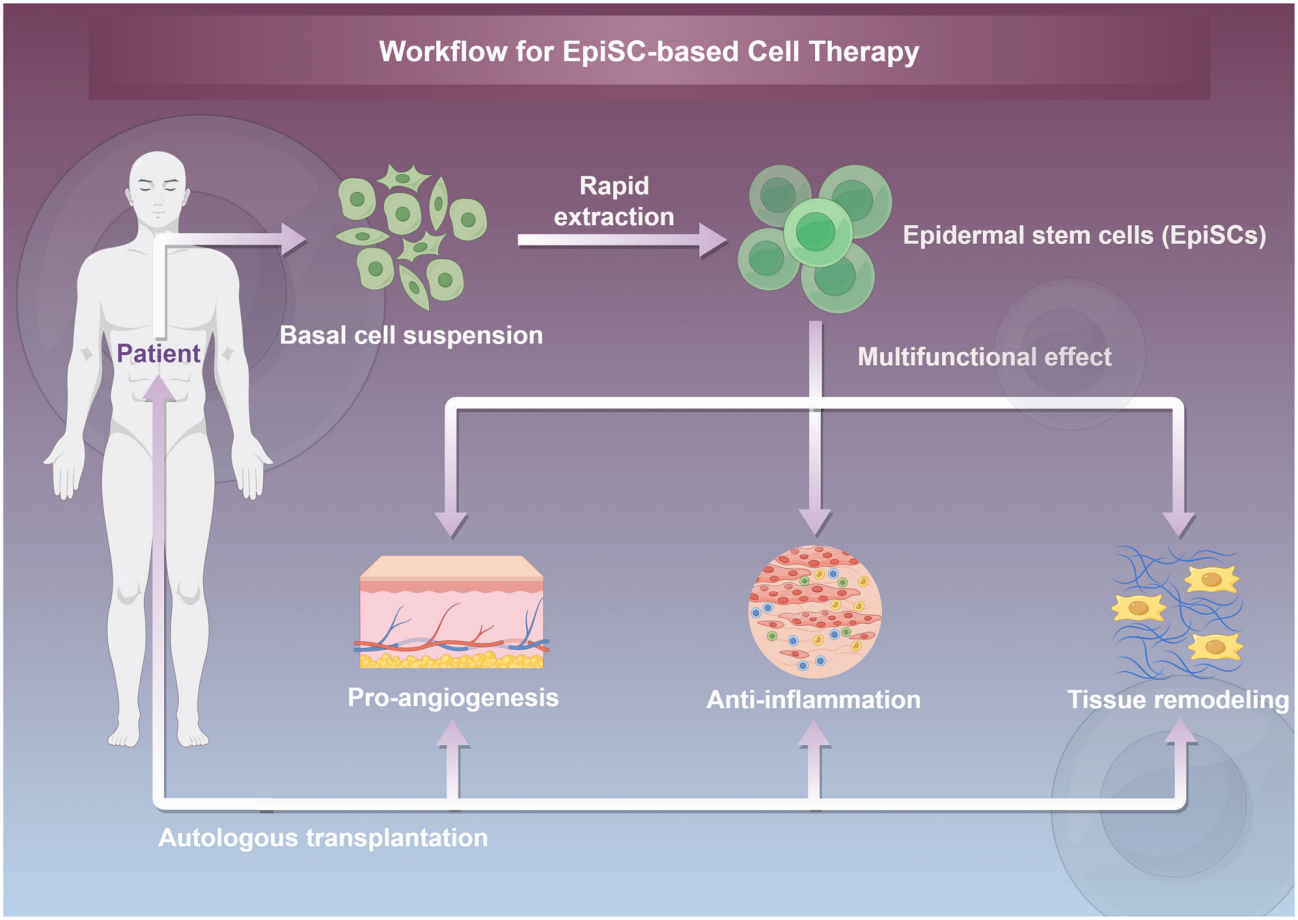
Workflow of autologous cell suspension enriched with EpiSCs for wound repair.

**FIGURE 2:**
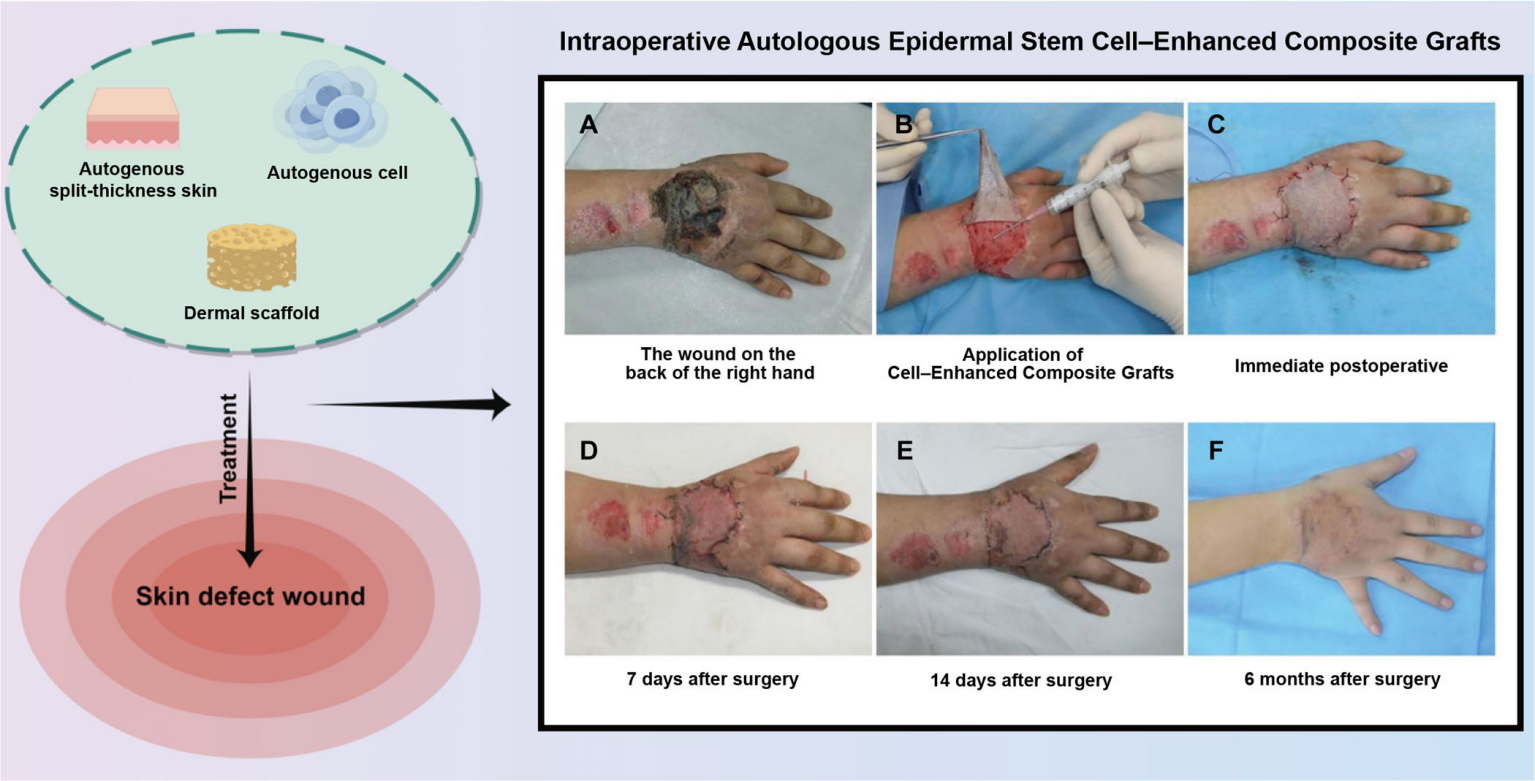
Representative therapeutic outcomes of wound repair using rapidly constructed tissue‐engineered skin based on autologous cell suspension enriched with EpiSCs. (A) A 23‐year‐old male patient presented with a residual wound on the dorsum of the right hand following a burn injury. (B) Wound repair using rapidly constructed tissue‐engineered skin. (C) Postoperative immediate photograph showing the wound site. (D) Photograph of the wound area 7 days post‐surgery. (E) Photograph of the wound area 14 days post‐surgery. (F) Photograph of the wound area 6 months post‐surgery.

Our research group has long focused on the therapeutic application of EpiSCs for wound healing [18, 19]. Early investigations suggested that autologous basal cell suspensions may significantly accelerate the repair of both acute and chronic wounds, with EpiSCs playing a central role (Figure 1) [20]. These cells are thought to promote angiogenesis, modulate immune responses, and support extracellular matrix remodeling, mainly through the release of exosomes and other bioactive substances [21, 22, 23]. Based on this discovery, we have developed a patented technology to rapidly obtain EpiSCs from split‐thickness skin and combine them with acellular allogeneic dermal matrices. After covering autologous split‐thickness skin graft (STSG), this allows for the rapid intraoperative preparation of tissue‐engineered skin, which has shown promising results in clinical applications (Figure 2).

To further investigate the mechanisms, we applied this rapidly constructed tissue‐engineered skin to repair full‐thickness skin wounds in rats. The results demonstrated that combining EpiSCs with acellular dermal matrix (ADM) significantly improved the angiogenesis process of skin grafts, enhancing the survival rate and healing effect of tissue‐engineered skin in vivo. Especially when using a combined strategy of ADM and autologous split‐thickness skin grafts, EpiSCs promoted angiogenesis, reduced skin graft contraction, and improved wound healing quality. Additionally, EpiSCs effectively promoted the regeneration of skin appendages such as hair follicles and sebaceous glands, thereby better reconstructing the structure and function of the skin. These findings further validate the potential of EpiSCs in repairing full‐thickness skin wounds [24].

This randomized controlled trial was therefore designed to evaluate the efficacy of intraoperatively prepared, EpiSCs‐enriched tissue‐engineered skin in the treatment of full‐thickness wounds. It was hypothesized that this strategy could improve wound healing outcomes, promote better graft integration, and reduce postoperative complications compared with conventional single‐stage composite grafting. The findings from this study may offer valuable insights into the clinical application of EpiSCs and their role in advancing regenerative strategies for complex skin defects.

## 2 Results

### 2.1 Patient Enrollment and Study Flow

**FIGURE 3:**
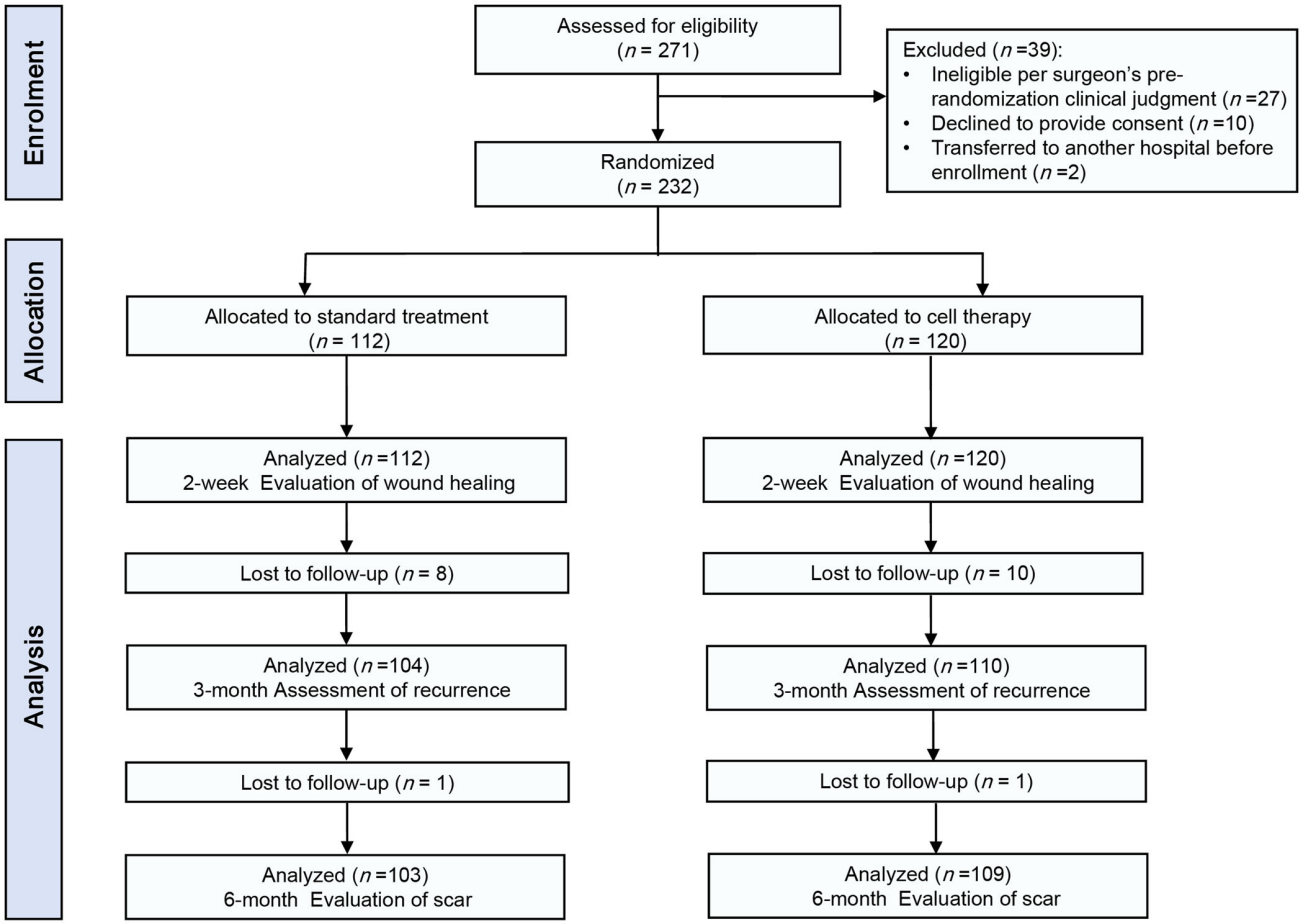
Clinical trial profile and study timeline.

As shown in (Figure 3), a total of 271 patients were assessed for eligibility, with 232 patients successfully enrolled in the study. These patients were then randomized into two groups: 112 patients received standard treatment, and 120 patients received the cell therapy. At the 6‐month follow‐up, scar evaluation was completed for 103 patients in the standard treatment group and 109 patients in the cell therapy group. The follow‐up period extended up to 6 months, with key assessments at 2 weeks, 3 months, and 6 months post‐treatment. Of the 232 randomized patients, all completed the 2‐week assessment and were included in the analysis at that time point. At the 3‐month follow‐up, 214 patients completed the evaluation and were analyzed, and at the 6‐month follow‐up, 212 patients completed the evaluation and were analyzed. Therefore, analyses were conducted on a per‐protocol (PP) basis, including only participants who completed the assigned treatment and follow‐up at each time point.

### 2.2 Baseline Characteristics of Patients

The baseline characteristics of patients in the standard treatment group (N = 112) and the cell therapy group (N = 120) were compared to ensure the groups were comparable prior to treatment. The analysis included gender distribution, age, cause of the wound, location of the wound, and wound area (Table 1).

**TABLE 1:** Baseline characteristics of patients receiving standard treatment versus cell therapy.

	Standard treatment (*N* = 112)	Cell therapy (*N* = 120)	*p*‐value
**Gender**			1.000
Male	70 (62.5%)	75 (62.5%)	
Female	42 (37.5%)	45 (37.5%)	
**Age (years)**			0.246
< 18	56 (50%)	53 (44.2%)	
18–60	49 (43.8%)	52 (43.3%)	
> 60	7 (6.2%)	15 (12.5%)	
**Cause of wound**			0.117
Scar	51 (45.5%)	48 (40%)	
Ulcer	29 (25.9%)	46 (38.3%)	
Burn	32 (28.6%)	26 (21.7%)	
**Location of wound**			0.118
Upper limb	48 (42.9%)	39 (32.5%)	
Lower limbs	52 (46.4%)	61 (50.8%)	
Trunk	10 (8.9%)	11 (9.2%)	
Head and neck	2 (1.8%)	9 (7.5%)	
**Area of wound (cm^2^)**			0.057
< 30	50 (44.6%)	55 (45.8%)	
30–50	27 (24.1%)	22 (18.3%)	
50–100	12 (10.7%)	27 (22.5%)	
> 100	23 (20.5%)	16 (13.3%)	

*Note*: Data are presented as *n* (%).

The gender distribution was identical between the two groups, with 62.5% male and 37.5% female in both the standard treatment and cell therapy groups. This indicates that gender was well balanced across the groups, with a *p*‐value of 1.000, showing no significant difference. Patients were divided into three age groups: under 18 years, 18–60 years, and over 60 years. In the standard treatment group, 50% were under 18 years, 43.8% were between 18 and 60 years, and 6.2% were over 60 years. In the cell therapy group, 44.2% were under 18 years, 43.3% were between 18 and 60 years, and 12.5% were over 60 years. While there were slightly more patients over 60 years in the cell therapy group, the difference was not statistically significant (*p* = 0.246). The causes of wounds included scars, ulcers, and burns. In the standard treatment group, 45.5% of wounds were due to scars, 25.9% to ulcers, and 28.6% to burns. In the cell therapy group, 40% of wounds were due to scars, 38.3% to ulcers, and 21.7% to burns. Although there was a higher proportion of ulcers in the cell therapy group, this difference was not statistically significant (*p* = 0.117). Wounds were categorized based on their location: upper limb, lower limbs, trunk, and head and neck. In the standard treatment group, 42.9% of wounds were on the upper limb, 46.4% on the lower limbs, 8.9% on the trunk, and 1.8% on the head and neck. In the cell therapy group, 32.5% of wounds were on the upper limb, 50.8% on the lower limbs, 9.2% on the trunk, and 7.5% on the head and neck. No statistically significant between‐group difference was detected in the overall distribution of wound location (*p* = 0.118). Wound areas were categorized as less than 30 cm^2^, 30–50 cm^2^, 50–100 cm^2^, and greater than 100 cm^2^. In the standard treatment group, 44.6% of wounds were smaller than 30 cm^2^, 24.1% were 30–50 cm^2^, 10.7% were 50–100 cm^2^, and 20.5% were larger than 100 cm^2^. In the cell therapy group, 45.8% of wounds were smaller than 30 cm^2^, 18.3% were 30–50 cm^2^, 22.5% were 50–100 cm^2^, and 13.3% were larger than 100 cm^2^. No statistically significant between‐group difference was detected in the overall distribution of wound area, although the global comparison was close to the conventional significance threshold (*p* = 0.057).

The baseline characteristics of patients in the standard treatment and cell therapy groups were generally comparable, with no statistically significant differences in gender, age, cause of wound, wound location, or wound area. However, trends were observed in the distribution of wound causes, locations, and sizes, which may warrant further consideration in the interpretation of treatment outcomes. These findings suggest that any differences observed in treatment efficacy are likely attributable to the interventions rather than baseline disparities between the groups.

### 2.3 Effect of Treatment on Wound Healing

A total of 232 patients were included in the study, with 141 achieving full wound healing within 2 weeks and 91 not fully healed (Table 2). The patients were divided into two groups based on the treatment method: standard treatment (*n* = 112) and cell therapy (*n* = 120).

**TABLE 2:** Univariable and multivariable analysis of factors influencing wound healing at 2 weeks.

Wound healing (2 weeks)	Not fully healed (*N* = 91)	Fully healed (*N* = 141)	OR (univariable)	OR (multivariable)
**Group**				
Standard treatment	51 (56%)	61 (43.3%)		
Cell therapy	40 (44%)	80 (56.7%)	1.67 (0.98–2.84, *p* = 0.058)	1.87 (1.06–3.28, *p* = 0.029)
**Gender**				
Male	65 (71.4%)	80 (56.7%)		
Female	26 (28.6%)	61 (43.3%)	1.91 (1.08–3.35, *p* = 0.025)	1.83 (1.02–3.27, *p* = 0.042)
**Age (years)**				
< 18	36 (39.6%)	73 (51.8%)		
18–60	46 (50.5%)	55 (39%)	0.59 (0.34–1.03, *p* = 0.064)	0.67 (0.36–1.26, *p* = 0.216)
> 60	9 (9.9%)	13 (9.2%)	0.71 (0.28–1.82, *p* = 0.479)	0.76 (0.26–2.19, *p* = 0.607)
**Cause of wound**				
Scar	34 (37.4%)	65 (46.1%)		
Ulcer	33 (36.3%)	42 (29.8%)	0.67 (0.36–1.23, *p* = 0.196)	0.78 (0.35–1.70, *p* = 0.529)
Burn	24 (26.4%)	34 (24.1%)	0.74 (0.38–1.44, *p* = 0.379)	0.80 (0.39–1.62, *p* = 0.529)
**Location of wound**				
Upper limb	28 (30.8%)	59 (41.8%)		
Lower limbs	49 (53.8%)	64 (45.4%)	0.62 (0.35–1.11, *p* = 0.108)	0.73 (0.37–1.43, *p* = 0.357)
Trunk	9 (9.9%)	12 (8.5%)	0.63 (0.24–1.68, *p* = 0.357)	0.62 (0.22–1.71, *p* = 0.355)
Head and neck	5 (5.5%)	6 (4.3%)	0.57 (0.16–2.03, *p* = 0.385)	0.53 (0.14–2.01, *p* = 0.350)
**Area of wound (cm^2^)**				
< 30	39 (42.9%)	66 (46.8%)		
30–50	23 (25.3%)	26 (18.4%)	0.67 (0.34–1.33, *p* = 0.249)	
50–100	13 (14.3%)	26 (18.4%)	1.18 (0.54–2.56, *p* = 0.672)	
> 100	16 (17.6%)	23 (16.3%)	0.85 (0.40–1.80, *p* = 0.670)	

*Note*: Data are presented as *n* (column percentage) to illustrate the compositional distribution of covariates within each outcome cohort.

Abbreviations: CI, confidence interval; OR, odds ratio.

At 2 weeks, complete wound healing was achieved in 80 of 120 patients in the cell therapy group and 61 of 112 patients in the standard treatment group, corresponding to healing rates of 66.7% and 54.5%, respectively. Representative wound‐healing progression in both treatment groups is shown in Figure S1. Univariable analysis showed a higher likelihood of complete healing in the cell therapy group (odds ratios [OR] = 1.67, 95% confidence interval [CI]: 0.98–2.84, *p* = 0.058), and this association reached statistical significance after adjustment (OR = 1.87, 95% CI: 1.06–3.28, *p* = 0.029). Gender was a significant predictor of wound healing. Females were more likely to achieve full wound healing compared to males. In the univariable analysis, the OR for females was 1.91 (95% CI: 1.08–3.35, *p* = 0.025). This finding remained significant in the multivariable analysis with an OR of 1.83 (95% CI: 1.02–3.27, *p* = 0.042). Age did not significantly affect the wound healing outcomes. Age was not significantly associated with complete wound healing. Compared with patients younger than 18 years, those aged 18–60 years showed a nonsignificant trend toward lower odds of complete healing in univariable analysis (OR = 0.59, 95% CI: 0.34–1.03, *p* = 0.064), which was attenuated after adjustment (OR = 0.67, 95% CI: 0.36–1.26, *p* = 0.216). This attenuation is likely attributable to confounding interactions with other demographic or treatment‐related variables within the adjusted model. The cause of the wound (scar, ulcer, or burn) did not significantly influence healing outcomes. Wound etiology was not significantly associated with complete healing. Compared with scar wounds, ulcers and burns were not associated with significantly different odds of healing in either univariable or multivariable analyses. Although forced into the multivariable model due to clinical relevance, the analysis confirmed no significant difference, yielding an OR of 0.78 (95% CI: 0.35–1.70, *p* = 0.529) for ulcers and an OR of 0.80 (95% CI: 0.39–1.62, *p* = 0.529) for burns, indicating no significant difference. The location of the wound also did not significantly impact the healing process. Wound location was not significantly associated with complete healing. In multivariable analysis, lower‐limb, trunk, and head‐and‐neck wounds did not differ significantly from upper‐limb wounds. Multivariable analysis confirmed the lack of significant association, with an OR of 0.73 (95% CI: 0.37–1.43, *p* = 0.357) for lower limb wounds compared to upper limb wounds. The area of the wound was not a significant factor in determining healing outcomes. Wound area was not significantly associated with complete healing in univariable analysis. Because none of the wound‐area categories approached the predefined *p* < 0.10 threshold, wound area was not included in the multivariable model. In the univariable analysis, the ORs were 0.67 (95% CI: 0.34–1.33, *p* = 0.249) for wounds 30–50 cm^2^, 1.18 (95% CI: 0.54–2.56, *p* = 0.672) for wounds 50–100 cm^2^, and 0.85 (95% CI: 0.40–1.80, *p* = 0.670) for wounds greater than 100 cm^2^. Because these values did not approach the predefined *p* < 0.10 threshold, wound area was excluded from the multivariable model.

In summary, the rapid construction of tissue‐engineered skin using EpiSCs demonstrated a significant positive effect on wound healing outcomes compared to standard treatment, particularly in female patients. Other factors, including age, wound cause, location, and size, were not significant predictors of healing success in this study.

### 2.4 Long‐Term Outcomes of Tissue‐Engineered Skin in Wound Healing and Recurrence

The study evaluated factors associated with wound recurrence at 3 months post‐treatment (Table 3). A total of 214 patients were assessed, with 196 (91.6%) experiencing no recurrence of wounds and 18 (8.4%) experiencing recurrence.

**TABLE 3:** Univariable and multivariable analysis of factors influencing wound recurrence at 3 months.

Wound healing (3 months)	No recurrence of wounds (*N* = 196)	Recurrence of wounds (*N* = 18)	OR (univariable)	OR (multivariable)
**Wound healing (2 weeks)**				
Not fully healed	66 (33.7%)	12 (66.7%)		
Fully healed	130 (66.3%)	6 (33.3%)	0.25 (0.09–0.71, *p* = 0.009)	0.26 (0.09‐0.75, *p* = 0.012)
**Group**				
Standard treatment	93 (47.4%)	11 (61.1%)		
Cell therapy	103 (52.6%)	7 (38.9%)	0.57 (0.21–1.54, *p* = 0.272)	
**Gender**				
Male	121 (61.7%)	10 (55.6%)		
Female	75 (38.3%)	8 (44.4%)	1.29 (0.49–3.42, *p* = 0.607)	
**Age (years)**				
< 18	99 (50.5%)	7 (38.9%)		
18–60	81 (41.3%)	8 (44.4%)	1.40 (0.49–4.02, *p* = 0.535)	0.79 (0.23–2.71, *p* = 0.711)
> 60	16 (8.2%)	3 (16.7%)	2.65 (0.62–11.33, *p* = 0.188)	1.23 (0.21–7.36, *p* = 0.821)
**Cause of wound**				
Scar	92 (46.9%)	5 (27.8%)		
Ulcer	56 (28.6%)	9 (50%)	2.96 (0.94–9.27, *p* = 0.063)	2.01 (0.48–8.39, *p* = 0.340)
Burn	48 (24.5%)	4 (22.2%)	1.53 (0.39–5.98, *p* = 0.538)	1.45 (0.34–6.10, *p* = 0.616)
**Location of wound**				
Upper limb	81 (41.3%)	5 (27.8%)		
Lower limbs	88 (44.9%)	12 (66.7%)	2.21 (0.75–6.54, *p* = 0.153)	1.68 (0.47–6.05, *p* = 0.428)
Trunk	18 (9.2%)	0 (0%)	0.00 (0.00–Inf, *p* = 0.992)	0.00 (0.00–Inf, *p* = 0.992)
Head and neck	9 (4.6%)	1 (5.6%)	1.80 (0.19–17.16, *p* = 0.609)	1.85 (0.18–18.66, *p* = 0.600)
**Area of wound (cm^2^)**				
< 30	87 (44.4%)	9 (50%)		
30–50	42 (21.4%)	4 (22.2%)	0.92 (0.27–3.16, *p* = 0.895)	
50–100	34 (17.3%)	2 (11.1%)	0.57 (0.12–2.77, *p* = 0.484)	
> 100	33 (16.8%)	3 (16.7%)	0.88 (0.22–3.45, *p* = 0.853)	

*Note*: Data are presented as *n* (column percentage) to illustrate the compositional distribution of covariates within each outcome cohort.

Abbreviations: CI, confidence interval; Inf, infinity; OR, odds ratio.

Patients who achieved full wound healing within the first 2 weeks had a significantly lower risk of wound recurrence at three months. Specifically, patients who achieved complete wound healing within the first 2 weeks had a lower 3‐month recurrence rate than those who did not achieve complete early healing (6/136 [4.4%] vs. 12/78 [15.4%]). Univariable analysis showed that early complete healing was associated with a significantly reduced risk of recurrence (OR = 0.25, 95% CI: 0.09–0.71, *p* = 0.009), and this association remained significant after adjustment (adjusted OR = 0.26, 95% CI: 0.09–0.75, *p* = 0.012). The study compared the effects of standard treatment versus tissue‐engineered skin therapy. Recurrence occurred in seven of 110 patients in the cell therapy group and 11 of 104 patients in the standard treatment group, corresponding to recurrence rates of 6.4% and 10.6%, respectively. Although numerically lower in the cell therapy group, this difference was not statistically significant (OR = 0.57, 95% CI: 0.21–1.54, *p* = 0.272). The lack of significance may suggest that while tissue‐engineered skin has potential benefits in reducing recurrence, further investigation with larger sample sizes is warranted. Gender was not significantly associated with wound recurrence in univariable analysis (OR = 1.29, 95% CI: 0.49–3.42, *p* = 0.607). Age was not a significant predictor of wound recurrence. Age was not a significant predictor of wound recurrence. In multivariable analysis, neither patients aged 18–60 years nor those older than 60 years differed significantly from patients younger than 18 years. Multivariable analysis showed no significant difference between age groups, with an OR of 0.79 (95% CI: 0.23–2.71, *p* = 0.711) for patients aged 18–60 years and an OR of 1.23 (95% CI: 0.21–7.36, *p* = 0.821) for those over 60 years. The cause of the wound, whether scar, ulcer, or burn, did not significantly impact recurrence. Wound etiology was not significantly associated with recurrence after adjustment. Although ulcers showed a nonsignificant trend toward higher odds of recurrence in univariable analysis (OR = 2.96, 95% CI: 0.94–9.27, *p* = 0.063), this association was attenuated in the multivariable model. The location of the wound did not significantly affect recurrence rates. Wound location was not significantly associated with recurrence. Lower‐limb wounds showed numerically higher odds of recurrence than upper‐limb wounds, but the difference was not statistically significant (OR = 1.68, 95% CI: 0.47–6.05, *p* = 0.428). Wound size was not a significant factor in predicting recurrence. Wound size was not significantly associated with recurrence in univariable analysis. Univariable analysis showed no significant differences across wound size categories, with an OR of 0.92 (95% CI: 0.27–3.16, *p* = 0.895) for wounds 30–50 cm^2^ and an OR of 0.88 (95% CI: 0.22–3.45, *p* = 0.853) for wounds larger than 100 cm^2^.

The rapid construction of tissue‐engineered skin using EpiSCs significantly reduced the risk of wound recurrence, particularly when early wound healing was achieved within the first 2 weeks. While the trend toward reduced recurrence with cell therapy was observed, it did not reach statistical significance, indicating the need for further research. Other factors such as gender, age, wound cause, location, and size did not significantly impact recurrence rates in this cohort. These findings highlight the potential of tissue‐engineered skin in improving long‐term outcomes in wound healing and support further investigation into its clinical applications.

### 2.5 Scar Assessment

The effectiveness of tissue‐engineered skin using EpiSCs in skin defect repair was evaluated by comparing scar characteristics between the standard treatment group (*N* = 103) and the cell therapy group (*N* = 109) at 6 months post‐treatment. The Vancouver Scar Scale (VSS) was used to assess four key parameters: pigmentation/color, height/thickness, vascularity, and pliability, as well as the total scar score (Table 4).

**TABLE 4:** Comparison of Vancouver Scar Scale scores between standard treatment and cell therapy groups.

VSS domain	Standard treatment (N = 103)	Cell therapy (N = 109)	*p*‐value
Pigmentation	1.0 (0.0–2.0)	0.0 (0.0–1.0)	0.001
Height	1.0 (1.0–2.0)	1.0 (0.0–1.0)	< 0.001
Vascularity	1.0 (1.0–2.0)	1.0 (0.0–1.0)	< 0.001
Pliability	2.0 (1.0–2.0)	1.0 (1.0–1.0)	< 0.001
Total VSS score	6.0 (4.0–8.0)	3.0 (2.0–4.0)	< 0.001

*Note*: Data are presented as median (interquartile range).

The cell therapy group exhibited significantly better outcomes in scar color, with a median score of 0.0 (interquartile range [IQR]: 0.0–1.0), compared to the standard treatment group, which had a median score of 1.0 (IQR: 0.0–2.0). The difference was statistically significant (*p* = 0.001), indicating a more favorable scar color in patients treated with cell therapy. Scar thickness was also significantly reduced in the cell therapy group, with a median score of 1.0 (IQR: 0.0–1.0), compared to a median score of 1.0 (IQR: 1.0–2.0) in the standard treatment group. The reduction in scar thickness with cell therapy was highly significant (*p* < 0.001). The vascularity scores were notably lower in the cell therapy group, with a median score of 1.0 (IQR: 0.0–1.0), compared to 1.0 (IQR: 1.0–2.0) in the standard treatment group. This difference was statistically significant (*p* < 0.001), suggesting that cell therapy leads to less vascularized scars. The cell therapy group showed a significant improvement in scar pliability, with a median score of 1.0 (IQR: 1.0–1.0), compared to 2.0 (IQR: 1.0–2.0) in the standard treatment group. The difference was statistically significant (*p* < 0.001), indicating that scars were more pliable in the cell therapy group. When considering the total scar score, which combines all four parameters, the cell therapy group had a median score of 3.0 (IQR: 2.0–4.0), significantly lower than the median score of 6.0 (IQR: 4.0–8.0) in the standard treatment group (*p* < 0.001). This comprehensive assessment demonstrates that cell therapy results in superior overall scar quality.

The results indicate that the rapid construction of tissue‐engineered skin using EpiSCs significantly improves scar outcomes in patients with skin defects. Compared to standard treatment, cell therapy leads to better scar color, reduced thickness, lower vascularity, and enhanced pliability, culminating in a significantly improved overall scar score. These findings highlight the potential of cell‐based therapies in enhancing the quality of wound healing and scar formation in clinical settings.

### 2.6 Safety Outcomes

No adverse events (AEs) or serious adverse events (SAEs) related to the interventions were observed during hospitalization or throughout the 6‐month follow‐up. These findings suggest that the treatment was well tolerated and safe in all patients.

## 3 Discussion

This study demonstrates the significant potential of EpiSCs in the construction of tissue‐engineered skin, particularly for enhancing wound healing and improving scar quality. Our findings provide compelling evidence that the integration of EpiSCs into advanced therapeutic modalities offers a promising alternative to standard treatment approaches for full‐thickness skin defects.

The most noteworthy outcome of this study is the significantly higher wound healing rate observed in the cell therapy group compared to the standard treatment group. The complete wound healing rate at 2 weeks was 66.7% in the cell therapy group and 54.5% in the standard treatment group, supporting the regenerative potential of EpiSC‐enhanced therapy. Multivariable analysis further showed that patients receiving EpiSC‐enhanced therapy had higher adjusted odds of achieving complete wound healing (adjusted OR = 1.87, 95% CI: 1.06–3.28, *p* = 0.029). Although age, gender, wound etiology, and wound location were controlled for in the analysis, it is worth noting that female patients demonstrated a higher likelihood of complete wound healing. This finding aligns with previous studies suggesting that biological differences between genders may influence wound healing processes [25, 26].

In addition to the improved healing rates, scar quality was markedly better in the cell therapy group, with significantly lower scores across multiple dimensions such as color, thickness, vascularization, and pliability. These results indicate that EpiSCs not only accelerate wound closure but also mitigate hypertrophic scarring, a major clinical concern [27, 28]. The superior scar outcomes are consistent with the known anti‐fibrotic properties of stem cells [29, 30], likely related to their ability to modulate the extracellular matrix and limit collagen accumulation. Importantly, reductions in VSS scores represent clinically meaningful improvements, reflecting reduced contracture and more natural appearance—factors directly linked to better functional recovery and cosmetic outcomes. These findings further support the therapeutic potential of stem cells in optimizing both function and aesthetics in wound repair.

Moreover, the absence of significant differences in healing outcomes based on wound location, size, or cause in our study is noteworthy. The consistent efficacy of EpiSCs‐based therapy across varying wound areas, including large defects (> 100 cm^2^), demonstrates that this approach is not restricted by wound dimensions. This suggests that the intraoperative application of EpiSCs may be broadly applicable across different types of wounds, making it a versatile and robust option for a wide range of complex clinical scenarios [31, 32].

Regarding recurrence, the 3‐month recurrence rate was numerically lower in the cell therapy group than in the standard treatment group (7/110 [6.4%] vs. 11/104 [10.6%]), but the difference did not reach statistical significance. This may be attributed to the relatively short follow‐up period of 6 months, which may not have been sufficient to capture long‐term recurrence patterns. Future studies with extended follow‐up are necessary to better understand the potential of EpiSCs in reducing wound recurrence over time. Despite the promising outcomes, the recurrence of wounds in a small subset of patients highlights the need for ongoing monitoring and optimization of the treatment protocols. While our study did not find a statistically significant difference in wound recurrence rates between the cell therapy and standard treatment groups, the observed trend suggests that further research with larger sample sizes and longer follow‐up periods is necessary to fully elucidate the long‐term benefits of EpiSCs‐based therapies. Importantly, beyond recurrence outcomes, our findings further support that EpiSC‐based therapy is not only effective in improving wound healing and scar quality, but also well tolerated without increasing the risk of treatment‐related AEs, consistent with previous clinical observations.

The results demonstrate that the intraoperative rapid preparation of EpiSCs‐based tissue‐engineered skin significantly enhances wound healing and scar quality, outperforming conventional composite skin grafting. Notably, improvements in scar appearance and VSS scores underscore its strong clinical potential. Given its dual benefits in function and aesthetics, this approach is particularly suitable for anatomically and cosmetically sensitive areas. Consistency with previous animal studies provides additional support for its clinical application [24]. Prior studies in burn patients have already highlighted the potential of epidermal cell–based therapies: for example, Gardien et al. reported that combining split‐thickness grafts with cultured epidermal cells accelerated re‐epithelialization and improved scar appearance and pliability at 12 months [33], while other series confirmed durable epidermal regeneration and scar improvement after cultured epithelial autografts in extensive burns. Similarly, in chronic nonhealing wounds, autologous non‐cultured epidermal or keratinocyte suspensions, as well as cultured keratinocyte grafts, have been shown to enhance closure rates and improve tissue quality [34, 35, 36, 37, 38, 39]. Compared to previous strategies, the key innovation of our approach lies in the intraoperative rapid sorting and immediate application of high‐purity autologous EpiSCs, which circumvents the need for prolonged in vitro culture. Unlike traditional methods that rely on cultured epidermal cells or keratinocyte grafts, our approach constructs tissue‐engineered skin that achieves bio‐inspired repair, replicating the structure and function of native skin. This method produces functional skin superior to conventional composite grafts, resulting in better wound healing and scar quality, particularly in functionally and cosmetically sensitive areas. By preserving the therapeutic advantages of EpiSCs, our approach significantly enhances clinical feasibility and accessibility [40]. Beyond clinical efficacy, the economic implications and resource utilization of this approach present a major translational advantage. By completely bypassing the exorbitant costs, specialized GMP facility requirements, and time delays associated with traditional in vitro cell expansion (such as Cultured Epidermal Autografts [CEA]), our intraoperative strategy significantly reduces overall medical resource consumption. While the cell preparation process necessitates certain specialized surgical consumables, completing the extraction and transplantation immediately within a single surgical stage minimizes the need for prolonged hospital stays and staged procedures, thereby substantially alleviating the overarching economic burden on patients and healthcare systems.

A common consideration in regenerative therapies is the theoretical risk of unchecked cell replication. In this study, we addressed this concern fundamentally through our methodological design. Biologically, our intervention is equivalent to an autologous skin graft at the cellular level. The autologous adult EpiSCs are derived directly from the patient's own healthy skin and applied immediately during the intraoperative setting, entirely without the addition of any exogenous growth factors. Because these cells are unmanipulated and not subjected to prolonged in vitro expansion, the inherent risks of unchecked proliferation—often associated with traditional cultured stem cells—are fundamentally eliminated. Consistent with this biological rationale, all healed wounds followed a normal physiological trajectory. We observed no clinical evidence of overgranulation, pathological tissue proliferation, or severe hypertrophic scarring in the cell therapy group during the follow‐up period. While the current six‐month follow‐up provides reassuring structural stability data, ongoing multicenter studies with extended long‐term observation will be valuable for further evaluating late‐stage scar maturation and overall aesthetic outcomes.

Several limitations merit consideration. First, despite rigorous protocol adherence via established SOPs, the single‐center design and prolonged enrollment period highlight the need for future multicenter trials. Additionally, while the sample size and six‐month follow‐up were adequate for primary endpoints, extended observation in larger cohorts is necessary to fully assess long‐term durability, functional integration, and late‐stage recurrence. Finally, regarding scar assessment, the VSS provided a highly standardized and clinically validated morphological baseline throughout our study. To effectively mitigate the inherent observer bias associated with its semi‐quantitative nature, all evaluations were rigorously performed independently by two blinded clinicians. Nevertheless, we acknowledge that VSS is primarily clinician focused. The absence of Patient‐Reported Outcome Measures (PROMs, such as the POSAS scale) to capture the subjective patient experience, along with the lack of objective biophysical data, remains a limitation of this current design. Integrating comprehensive PROMs into our future prospective, multicenter trials will be essential to provide a multidimensional assessment, refine clinical interpretation, and optimize broader clinical adoption.

## 4 Methods

### 4.1 Study Design and Participants

This prospective, randomized controlled trial was conducted at the First Affiliated Hospital of Sun Yat‐sen University between January 2014 and June 2023 to assess the efficacy of rapidly constructed EpiSC‐based tissue‐engineered skin in the repair of skin defects. A total of 232 patients were randomly assigned to receive either standard treatment (*n* = 112) or cell therapy (*n* = 120). To preserve statistical independence, only one target wound per patient was included for randomization and follow‐up. In patients with multiple eligible wounds, the wound expected to provide the greatest overall clinical benefit was selected. The study protocol (Supporting Information Material 1) was approved by the Medical Ethics Committee of the First Affiliated Hospital of Sun Yat‐sen University (Approval No. [2013]151), and written informed consent was obtained from all participants. The trial was registered at ClinicalTrials.gov (NCT02070809).

### 4.2 Ethical Considerations

The study protocol was approved by the institutional review board. All participants provided written informed consent, which included disclosure that the technology used in this study is subject to a patent held by the investigators. Participants were informed that their decision to participate would not affect their access to standard treatment.

### 4.3 Randomization and Blinding

Participants were randomly assigned in a 1:1 ratio to the standard treatment group or the cell therapy group. The randomization sequence was generated using a computer‐based algorithm with a permuted block randomization method, implemented in SPSS software (version 26.0, IBM Corp., Armonk, NY, USA). The sequence was produced by an independent statistician who was not involved in patient recruitment, treatment, or outcome assessment. Allocation concealment was ensured through the use of sequentially numbered, opaque, sealed envelopes, which were opened only after the patient had been deemed eligible and enrolled.

Blinding was maintained for both participants and outcome assessors. Patients were unaware of their treatment allocation, and the investigators responsible for evaluating wound healing, recurrence, and scar outcomes were blinded to group assignment. Treating clinicians could not be blinded due to the nature of the interventions; however, they did not participate in outcome assessments. These measures were implemented to minimize both selection bias and assessment bias, ensuring the integrity of the trial results.

### 4.4 Inclusion Criteria

Eligible patients were those requiring skin grafting for full‐thickness skin defects caused by burns, ulcers, scars, or other conditions; had a wound area greater than 9 cm^2^; were willing to participate in the clinical trial and provide written informed consent; and were mentally capable of understanding and following medical instructions.

### 4.5 Exclusion Criteria

Patients were excluded if they had a known allergy to trypsin or collagen; acute systemic infection, uncontrolled severe disease, or serious organ dysfunction; planned pregnancy during the study period or within 6 months after skin grafting; malignant tumors, autoimmune diseases, or use of high‐dose glucocorticoids, defined as ≥ 40 mg prednisone per day or an equivalent dose for ≥ 2 weeks; positive bacterial culture indicating wound infection; limb vascular neuropathy; an expected inability to survive during the study period; or participation in another clinical study within the previous 12 weeks.

### 4.6 Interventions

Once a viable wound bed suitable for grafting had been established, surgical treatment was performed on (1) wounds with healthy granulation tissue formed after thorough debridement of burns or ulcers; and (2) surgically created fresh wounds following the excision of extensive scars or contracture release, where direct closure was not feasible due to tissue loss and skin grafting was required to achieve wound closure. Both the cell therapy group and the standard treatment group underwent a single‐stage surgical procedure.

Both groups underwent standardized STSG harvesting from donor sites, primarily the scalp or thighs, selected according to skin color matching and tissue availability, with a uniform graft thickness of 0.25 mm. In the standard treatment group, after wound bed preparation, the ADM was applied and immediately covered with the STSG. In the cell therapy group, the longest diameter and maximum width of the wound were measured using a sterile surgical ruler. Subsequently, a rectangular donor skin area was designed based on these measurements, and the same design method was used in the standard treatment group. An STSG was then harvested from this rectangular donor area using an electric dermatome. The length and width of the donor area were adjusted according to wound morphology to ensure that, after contour trimming of the graft, sufficient residual graft fragments corresponding to approximately ≥ 2.5% of the original wound area were available for cell preparation. These residual fragments were processed intraoperatively using the MYSEED kit (Guangzhou Myseed Medical Technology Co., Ltd., Guangzhou, China), a standardized cell isolation system, to obtain an EpiSC‐enriched basal cell suspension. The resulting cell suspension was prepared for immediate intraoperative application. During the grafting phase, approximately 1 mL of the suspension was allocated for every 35–45 cm^2^ of the target area. The suspension was first sprayed onto the wound bed, followed by coverage with ADM. A second spray was applied onto the ADM surface, and finally, the STSG was overlaid. Postoperative care was identical for both groups: initial dressing changes were performed on Day 6, followed by daily or every‐other‐day changes based on graft adherence, continuing until complete healing was achieved.

### 4.7 Outcome Measures

The primary outcome was scar quality, which was assessed using the VSS [41]. The scale consists of four items: pigmentation, vascularity, pliability, and height. Each item was evaluated and scored independently by two experienced, blinded clinicians to minimize observer bias, and the average scores were recorded for analysis. The total score ranges from 0 to 13 points, with lower scores indicating better scar quality and higher scores indicating poorer scar quality. Secondary outcomes included complete wound healing at 2 weeks and wound recurrence at 3 months post‐treatment. Complete healing was defined as complete epithelialization of the wound with no signs of infection or inflammation. Recurrence was defined as reopening or breakdown of the previously healed target wound within 3 months after treatment.

### 4.8 Safety Assessment

AEs and SAEs unrelated to the predefined efficacy outcomes were monitored throughout hospitalization and at follow‐up visits (2 weeks, 3 months, and 6 months). Particular attention was given to allergic reactions to the EpiSCs preparation or ADM, systemic symptoms, and laboratory abnormalities. All AEs/SAEs were documented and assessed for severity and causality by the investigators, and reviewed by an independent monitoring committee.

### 4.9 Sample Size Calculation

The sample size was calculated prior to the trial based on an expected improvement in scar quality with cell therapy. Using a two‐sided test with a significance level (α) of 0.05 and a power (1 − β) of 80% to detect a significant difference in the VSS scores between the two groups, the calculated sample size required was approximately 198 patients (99 per group). Accounting for an anticipated dropout rate of 15% during the 6‐month follow‐up period, approximately 233 patients would be required (198 ÷ 0.85 = 232.9). Ultimately, 232 patients were randomized, and 212 completed the 6‐month evaluation, exceeding the originally calculated minimum of 198 evaluable patients.

### 4.10 Statistical Analysis

Statistical analyses were performed using SPSS software (version 26.0, IBM Corp., Armonk, NY, USA). Baseline characteristics between groups were compared using the chi‐square test for categorical variables and the Mann–Whitney *U* test for continuous variables. VSS scores were compared between groups using the Mann–Whitney *U* test due to their non‐normal distribution.

Binary logistic regression models were constructed to identify factors associated with wound healing at 2 weeks and wound recurrence at 3 months. Candidate variables included treatment group, sex, age, wound etiology, wound location, wound area, and wound healing status at 2 weeks (for the 3‐month recurrence model only). Variables with *p* < 0.10 in univariable analysis were considered for inclusion in the multivariable model. Additionally, clinically important covariates known to influence wound healing outcomes—namely age, wound etiology, and wound location—were forced into the multivariable model regardless of their univariable significance levels to adjust for potential confounding. Multivariable analyses were performed using the Enter method, and adjusted ORs with 95% CIs were reported.

Analyses were primarily conducted on a PP basis, including only participants who completed the assigned treatment and follow‐up. Missing data were handled by listwise deletion. Continuous variables are presented as medians with IQR. A two‐sided *p* < 0.05 was considered statistically significant.

## Author Contributions

**Zhicheng Hu**: conceptualization, data curation, methodology, project administration, writing – original draft. **Hao Yang**: Data curation, formal analysis, methodology, writing – original draft. **Peng Wang**: conceptualization, data curation, project administration, writing – original draft. **Xiaoling Cao**: formal analysis, methodology, writing – original draft. **Peng Wang**: formal analysis, methodology, writing – original draft. **Bing Tang**: data curation, methodology. **Hailin Xu**: data curation, writing – review and editing. **Zhiyong Wang**: formal analysis, methodology, writing – review and editing. **Shaobin Huang**: methodology, writing – review and editing. **Jian Zhang**: data curation, writing – review and editing. **Chunli Xue**: data curation, writing – review and editing. **Qiang Zhou**: data curation, project administration, writing – review and editing. **Xiaohui Li**: writing – review and editing. **Honglin Wu**: writing – review and editing. **Yi Zhang**: supervision, writing – review and editing. **Julin Xie**: supervision, writing –review and editing. **Jiayuan Zhu**: supervision, funding acquisition, writing – review and editing. All authors have read and approved the final manuscript.

## Funding

This study was funded by the Sun Yat‐sen University Clinical Research 5010 Program (2013001, 2017013), the National Natural Science Foundation of China (82572883), the China Postdoctoral Science Foundation (2025M772347), the Guangdong Provincial Natural Science Foundation of China (2024A1515010485, 2026A1515010501, 2026A1515010461), the Clinical Major Technology Project of Guangzhou Municipal Health Commission (2026P‐ZD025), the High‐level Clinical Research Program of the First Affiliated Hospital of Sun Yat‐sen University, and the Fundamental Research Program of Shanxi Province (202303021212368).

## Ethics Statement

The study protocol was approved by the institutional ethics committee (Approval No. [2013]151). Informed consent was obtained from all participants in the study. The clinical trial was registered at ClinicalTrials.gov (Registration number: NCT02070809; https://clinicaltrials.gov).

## Conflicts of Interest

One or more authors hold a patent related to the technology described in this study. The details of this patent are disclosed here. Other authors have no conflicts of interest.

## Supporting information




**Supporting File**: mco270973‐sup‐0001‐SuppMat.docx.

## Data Availability

Data used to support the findings of this study are available from the corresponding author upon reasonable request.
