# Supplementary material for: Bio‐Inspired Skin Reconstruction Using Intraoperative Autologous Epidermal Stem Cell–Enhanced Composite Grafts: A Randomized Trial
Source: MedComm (2020). 2026 Sep 6;7(9):e70973. doi: 10.1002/mco2.70973 (PMC13547091; doi:10.1002/mco2.70973)
Supplement: Supplementary file 1 [file MCO2-7-e70973-s001.docx]

**Bio-Inspired Skin Reconstruction Using Intraoperative Autologous Epidermal Stem Cell–Enhanced Composite Grafts: A Randomized Trial**

Zhicheng Hu^1,#^, Hao Yang^1,#^, Peng Wang^1,10,#^, Xiaoling Cao^1,#^, Peng Wang^1,#^, Bing Tang^1^, Hailin Xu^3^, Zhiyong Wang^4^, Shaobin Huang^5^, Jian Zhang^6^, Chunli Xue^7^, Qiang Zhou^8^, Xiaohui Li^9^, Honglin Wu^1^, Yi Zhang^2,*^, Julin Xie^1,*^, Jiayuan Zhu^1,*^

#Zhicheng Hu, Hao Yang, Peng Wang(affiliations 1 and 10), Xiaoling Cao and Peng Wang(affiliation 1) contributed equally to this work.

^1^First Affiliated Hospital of Sun Yat-sen University, Department of Burn and Wound Repair, Guangzhou, China, 510080

^2^Affiliated Hospital of Nantong University, Department of Burn and Plastic Surgery, Department of Wound Repair Surgery, Nantong, China, 226001

^3^Dermatology Hospital of Southern Medical University, Department of Dermatology, Guangzhou, China, 510091

^4^The Third Affiliated Hospital of Guangzhou Medical University, Department of Joint Surgery, Guangzhou, China, 510150

^5^Sixth Affiliated Hospital of Sun Yat-sen University Department of Plastic and Cosmetic Surgery, Guangzhou, China, 510510

^6^Sun Yat-Sen Memorial Hospital of Sun Yat-Sen University, Department of Plastic and Reconstructive Surgery, Guangzhou, China, 510000

^7^Huizhou Central People's Hospital, Department of Burn Surgery, Huizhou, China, 516001

^8^The First People's Hospital of Zhaoqing, Department of Burn and Plastic Surgery, Zhaoqing, China, 526000

^9^Shenzhen Hospital，Southern Medical University，Department of Plastic Surgery & Burn Surgery，Shenzhen，China，518101

^10^Linfen Central Hospital, Department of Burns and Plastic Surgery, Linfen, China, 041000

***Corresponding authors:**

Dr Jiayuan Zhu, First Affiliated Hospital of Sun Yat-sen University, Guangzhou, China (E-mail: zhujiay@mail.sysu.edu.cn),

Dr Julin Xie, First Affiliated Hospital of Sun Yat-sen University, Guangzhou, China (E-mail: xiejulin@mail.sysu.edu.cn),

Dr Yi Zhang, Department of Burn and Plastic Surgery, Department of Wound Repair Surgery, Affiliated Hospital of Nantong University, Nantong, China (E-mail: zhangyi@ntu.edu.cn).

**Figure S1. Representative clinical outcomes in patients receiving standard treatment and cell therapy.**

Representative clinical photographs showing wound status before surgery, during surgery, 2 weeks after surgery, and 6 months after surgery in the standard treatment and cell therapy groups.

**Supplementary Materials - Index**

| **Supplementary Methods** |  |
| --- | --- |
| Trial Design, Settings & Eligibility Criteria | Pag. 3 |
| Research Process | Pag. 3-5 |
| Clinical Outcomes | Pag. 5-6 |
| Sample Size & Randomization | Pag. 6 |
| Statistical Analysis | Pag. 6-7 |
| Ethics & Others | Pag. 7 |

Supplementary Methods

**I Trial Design, Settings & Eligibility Criteria**

**1.1 Study Design and Participants**

This study protocol describes a prospective, randomized controlled trial conducted at the First Affiliated Hospital of Sun Yat-sen University between January 2014 and June 2023 to assess the efficacy of rapidly constructed epidermal stem cell (EpiSC)-based tissue-engineered skin in the repair of skin defects. Eligible participants were to be allocated in a 1:1 ratio to receive either standard treatment or cell therapy. To preserve statistical independence, only one target wound per patient was included for randomization and follow-up. In patients with multiple eligible wounds, the target wound was selected before randomization according to the prespecified clinical assessment. The protocol was approved by the Medical Ethics Committee of the First Affiliated Hospital of Sun Yat-sen University (Approval No. [2013]151), and written informed consent was obtained before enrollment. The trial was registered at ClinicalTrials.gov (NCT02070809).

**1.2 Inclusion Criteria**

Eligible patients were those who required skin grafting for full-thickness skin defects caused by burns, ulcers, scars, or other conditions; who had a wound area greater than 9 cm²; who were willing to participate in the clinical trial, provide written informed consent (with consent from legal guardians for minors), and comply with follow-up requirements; and who were mentally capable of understanding and following medical instructions.

**1.3 Exclusion Criteria**

Patients were excluded if they had a known allergy to trypsin or collagen; acute systemic infection, uncontrolled severe disease, or serious organ dysfunction; planned pregnancy during the study period or within 6 months after skin grafting; malignant tumors, autoimmune diseases, or use of high-dose glucocorticoids, defined as ≥40 mg prednisone per day or an equivalent dose for ≥2 weeks; positive bacterial culture indicating wound infection; limb vascular neuropathy; an expected inability to survive during the study period; or participation in another clinical study within the previous 12 weeks.

**1.4 Withdrawal Criteria**

Participants could withdraw at the request of the patient or legal guardian, if life-threatening adverse events occurred, or if follow-up was lost for two consecutive scheduled visits.

**II Research Process**

**2.1 Patient Screening Period (Day -7 to Day 0; Visit 1)**

① Informed consent signing; ② medical history review; ③ basic personal information; ④ current medication history and treatment status; ⑤ routine physical examination, including height, weight, and vital signs; ⑥ physical examination, including specialized examination of wounds/scars; ⑦ wound bacterial culture and drug sensitivity testing; ⑧ complete blood count, urinalysis, biochemical liver and kidney function tests, coagulation profile, preoperative screening panel, and serum pregnancy test when applicable.

**2.2 Patient Enrollment (Day 1; Visit 2)**

At enrollment, past/concomitant medications and any pre-randomization adverse events were reviewed and recorded in the Case Report Form (CRF). After eligibility was confirmed, participants were assigned in a 1:1 ratio to the standard treatment group or the cell therapy group as described in Section 4.2.

**2.3 Interventions**

Surgical treatment was performed after a viable wound bed suitable for grafting had been established, including (1) wounds with healthy granulation tissue formed after thorough debridement of burns or ulcers; and (2) surgically created fresh wounds following the excision of extensive scars or contracture release, where direct closure was not feasible due to tissue loss and skin grafting was required to achieve wound closure. Both the cell therapy group and the standard treatment group underwent a single-stage surgical procedure.

Both groups underwent standardized STSG harvesting from donor sites, primarily the scalp or thighs, selected according to skin color matching and tissue availability, with a uniform graft thickness of 0.25 mm. In the standard treatment group, after wound bed preparation, the acellular dermal matrix (ADM) was applied and immediately covered with the STSG. In the cell therapy group, the longest diameter and maximum width of the wound were measured using a sterile surgical ruler. Subsequently, a rectangular donor skin area was designed based on these measurements, and the same design method was used in the standard treatment group. An STSG was then harvested from this rectangular donor area using an electric dermatome. The length and width of the donor area were adjusted according to wound morphology to ensure that, after contour trimming of the graft, sufficient residual graft fragments corresponding to approximately ≥2.5% of the original wound area were available for cell preparation. These residual fragments were processed intraoperatively using the MYSEED® kit (Guangzhou Myseed Medical Technology Co., Ltd., Guangzhou, China), a standardized cell isolation system, to obtain an epidermal stem cell (EpiSC)-enriched basal cell suspension. The resulting cell suspension was prepared for immediate intraoperative application. During the grafting phase, approximately 1 mL of the suspension was allocated for every 35–45 cm² of the target area. The suspension was first sprayed onto the wound bed, followed by coverage with ADM. A second spray was applied onto the ADM surface, and finally, the STSG was overlaid. Postoperative care was identical for both groups: initial dressing changes were performed on day 6, followed by daily or every-other-day changes based on graft adherence, continuing until complete healing was achieved.

**2.4 Postoperative Follow-up**

Postoperative follow-up assessments were scheduled at 2 weeks, 3 months, and 6 months after treatment. At 2 weeks, complete wound healing was assessed and standardized wound photographs were retained when available. At 3 months, wound recurrence was assessed. At 6 months, scar quality was assessed using the Vancouver Scar Scale (VSS). Adverse events and concomitant medications were recorded throughout hospitalization and at all follow-up visits.

**2.5 Safety Assessment**

Adverse events (AEs) and serious adverse events (SAEs) unrelated to the predefined efficacy outcomes were monitored throughout hospitalization and at follow-up visits (2 weeks, 3 months, and 6 months). Particular attention was given to allergic reactions to the EpiSCs preparation or acellular dermal matrix, systemic symptoms, laboratory abnormalities, graft failure, infection requiring additional treatment, and any re-operation related to the treated wound. SAEs included death, life-threatening events, hospitalization or prolonged hospitalization, persistent or significant disability/incapacity, amputation, re-operation, or other medically important events. All AEs/SAEs were documented and assessed for severity and causality by the investigators and reviewed by an independent monitoring committee.

**III Clinical Outcomes**

**3.1 Primary Outcome**

The primary outcome was scar quality at 6 months, which was assessed using the Vancouver Scar Scale (VSS). The scale consists of four items: pigmentation, vascularity, pliability, and height. Each item was evaluated and scored independently by two experienced, blinded clinicians to minimize observer bias, and the average scores were recorded for analysis. The total score ranges from 0 to 13 points, with lower scores indicating better scar quality and higher scores indicating poorer scar quality.

**3.2 Secondary Outcomes**

Secondary outcomes included complete wound healing at 2 weeks and wound recurrence at 3 months post-treatment. Complete healing was defined as complete epithelialization of the wound with no signs of infection or inflammation. Recurrence was defined as reopening or breakdown of the previously healed target wound within 3 months after treatment.

**3.3 Safety Outcomes**

Safety outcomes included adverse events and serious adverse events, with particular attention to allergic reactions to the EpiSCs preparation or acellular dermal matrix, systemic symptoms, and laboratory abnormalities.

**3.4 Quality Control and Data Management**

Study data were recorded in CRFs/eCRFs and archived securely. Source documents, protocol deviations, missing data, and follow-up status were documented according to institutional requirements. Outcome assessors were blinded to group assignment, and AEs/SAEs were reviewed by an independent monitoring committee.

**IV Sample Size & Randomization**

**4.1 Sample Size Calculation**

The sample size was calculated prior to the trial based on an expected improvement in scar quality with cell therapy. Using a two-sided test with a significance level (α) of 0.05 and a power (1-β) of 80% to detect a significant difference in the Vancouver Scar Scale scores between the two groups, the calculated sample size required was approximately 198 patients (99 per group). Accounting for an anticipated dropout rate of 15% during the 6-month follow-up period, approximately 233 patients would be required (198÷0.85=232.9).

**4.2 Randomization and Blinding**

Participants were assigned in a 1:1 ratio to the standard treatment group or the cell therapy group. The randomization sequence was generated using a computer-based algorithm with a permuted block randomization method, implemented in SPSS software (version 26.0, IBM Corp., Armonk, NY, USA). The sequence was produced by an independent statistician who was not involved in patient recruitment, treatment, or outcome assessment. Allocation concealment was ensured through the use of sequentially numbered, opaque, sealed envelopes, which were opened only after the patient had been deemed eligible and enrolled.

Blinding was maintained for both participants and outcome assessors. Patients were unaware of their treatment allocation, and the investigators responsible for evaluating wound healing, recurrence, and scar outcomes were blinded to group assignment. Treating clinicians could not be blinded due to the nature of the interventions; however, they did not participate in outcome assessments. These measures were implemented to minimize both selection bias and assessment bias.

**V Statistical Analysis**

Statistical analyses were prespecified and performed using SPSS software (version 26.0, IBM Corp., Armonk, NY, USA). Baseline characteristics between groups were compared using the chi-square test for categorical variables and the Mann–Whitney U test for continuous variables. Vancouver Scar Scale scores were compared between groups using the Mann–Whitney U test due to their non-normal distribution.

Binary logistic regression models were prespecified to identify factors associated with wound healing at 2 weeks and wound recurrence at 3 months. Candidate variables included treatment group, sex, age, wound etiology, wound location, wound area, and wound healing status at 2 weeks (for the 3-month recurrence model only). Variables with P < 0.10 in univariable analysis were considered for inclusion in the multivariable model. Additionally, clinically important covariates known to influence wound healing outcomes—namely age, wound etiology, and wound location—were forced into the multivariable model regardless of their univariable significance levels to adjust for potential confounding. Multivariable analyses were performed using the Enter method, and adjusted odds ratios (ORs) with 95% confidence intervals (CIs) were reported.

Analyses were planned to be conducted primarily on a per-protocol (PP) basis, including only participants who completed the assigned treatment and follow-up. Missing data were handled by listwise deletion. Continuous variables are presented as medians with interquartile ranges (IQR). A two-sided P < 0.05 was considered statistically significant.

**VI Ethics & Others**

**6.1 Ethical Approval**

The study protocol was approved by the institutional ethics committee (Approval No. [2013]151). The clinical trial was registered at ClinicalTrials.gov (Registration number: NCT02070809).

**6.2 Informed Consent**

Written informed consent was obtained from all participants before enrollment. The informed consent included disclosure that the technology used in this study is subject to a patent held by the investigators. Participants were informed that their decision to participate would not affect their access to standard treatment. For minors, consent was provided by legal guardians according to institutional requirements.

**6.3 Data Management and Confidentiality**

Study records, CRFs/eCRFs, source documents, and follow-up data were stored securely with access limited to authorized study personnel. De-identified data may be made available from the corresponding author upon reasonable request, subject to institutional and ethical requirements.

**6.4 Adverse Event Reporting**

All AEs/SAEs were documented and assessed for severity and causality by the investigators and reviewed by an independent monitoring committee. Serious adverse events were reported according to institutional and ethics committee requirements.
